# A call for caution in analysing mammalian co-transfection experiments and implications of resource competition in data misinterpretation

**DOI:** 10.1038/s41467-021-22795-9

**Published:** 2021-05-05

**Authors:** Roberto Di Blasi, Masue M. Marbiah, Velia Siciliano, Karen Polizzi, Francesca Ceroni

**Affiliations:** 1 Department of Chemical Engineering, Imperial College London, South Kensington Campus, London, UK; 2 Imperial College Centre for Synthetic Biology, South Kensington Campus, London, UK; 3 Synthetic and Systems Biology lab for Biomedicine, Istituto Italiano di Tecnologia-IIT, Largo Barsanti e Matteucci, Naples (ITA), Italy

**Keywords:** Synthetic biology, Biomedical engineering

## Abstract

Transient transfections are routinely used in basic and synthetic biology studies to unravel pathway regulation and to probe and characterise circuit designs. As each experiment has a component of intrinsic variability, reporter gene expression is usually normalized with co-delivered genes that act as transfection controls. Recent reports in mammalian cells highlight how resource competition for gene expression leads to biases in data interpretation, with a direct impact on co-transfection experiments. Here we define the connection between resource competition and transient transfection experiments and discuss possible alternatives. Our aim is to raise awareness within the community and stimulate discussion to include such considerations in future experimental designs, for the development of better transfection controls.

## Resource competition in engineered hosts

**Fig. 1:**
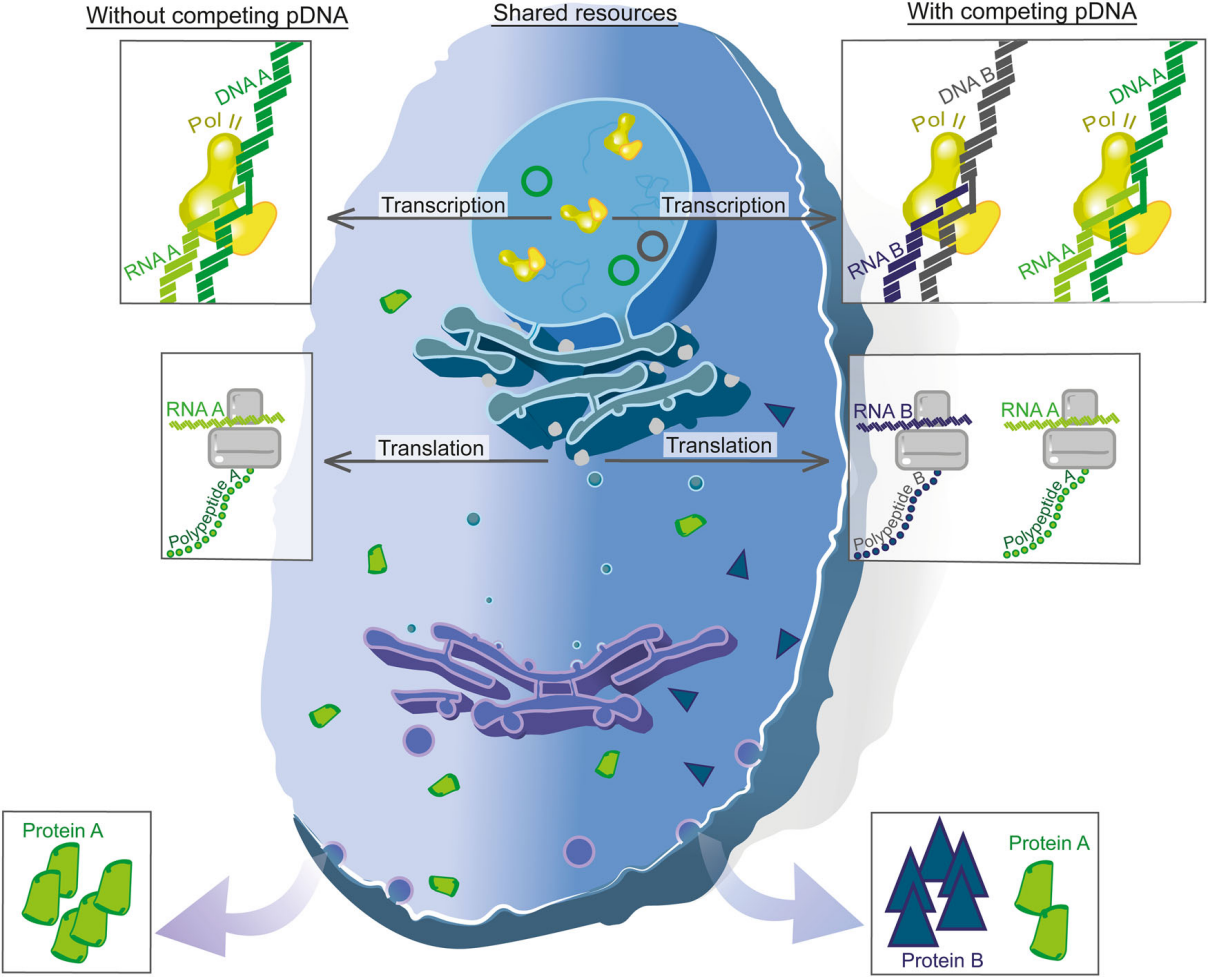
A comparison of resource allocation in mammalian cells upon transfection of a genetic construct with and without a co-expressed control. Two scenarios are shown in parallel where a plasmid (pDNA) harbouring Protein A is transfected alone (left) or co-transfected with Protein B (right). In the nucleus, pDNA are processed by a finite number of RNA polymerase II (Pol II) molecules; therefore heterologous (A and B) and endogenous genes must compete for its availability. In a scenario with greater competition (right), less mRNA is transcribed from DNA A compared to a scenario with less competition (left). To compound this issue, cells also contain a finite number of ribosomes. As such, competition for translational machinery leads to unpredictable production of A and B proteins.

Expression of foreign DNA in microbes or mammalian cells for the expression of proteins of interest is common practice in molecular, cellular and synthetic biology for the study of gene function (foundational research) or for the production of new therapeutics or commercial biologics (translational research). Pioneering work in bacteria unveiled that cells possess a finite amount of intracellular resources available for gene expression^1^ (e.g. RNA polymerases, ribosomes, proteases, energy sources) and competition for the utilisation of such resources arises between exogenous genes, as well as between exogenous and endogenous genes. Resource competition implies that if one transcriptional unit utilises more resources, this leads to a decrease in the resources available to operate cellular physiological processes or to express other synthetic transcriptional units, leading to an artificial decrease in their expression levels (Fig. 1). Thus, expression cassettes, otherwise independently regulated, become interlinked by resource usage. Gyorgy et al. demonstrated that competition for gene expression resources between two independent transcriptional units causes a counterintuitive coupling in their expression in *E. coli*^2^. The authors transformed *E. coli* with a plasmid bearing a constitutive GFP expression cassette and an AHL-inducible RFP expression cassette and observed how increasing inducer concentration led to increased RFP, but decreased GFP expression, despite the two proteins being functionally independent. Recently, Ceroni et al. quantified how the presence of synthetic constructs can cause decreased growth, global transcriptional rearrangements and emergence of escape mutants in engineered *E. coli* populations^3,4^. In mammalian cells Huliak et al. observed interference between P_CMV_-driven EGFP expression and other co-transfected luciferase-expressing promoters^5^. The authors reported a dramatic decrease in luminescence following co-transfection of the P_CMV_-EGFP plasmid with increasing amounts of the latter leading to more drastic interference. Since different promoters displayed the same behaviour, resource competition was suggested to be the likely cause of the phenomenon.

More recently, resource competition in mammalian cells has been quantified and shown to undermine the performance of genetic circuits, prompting a deeper investigation of the issue^6–8^. Frei et al. demonstrated that, at very least, both transcription and translation are affected by intracellular resource availability^7^. In a first pilot experiment, the authors showed that co-transfection of two functionally independent plasmids, one bearing a constitutively expressed reporter and the other a fluorescent protein under the control of doxycycline (Dox) via the TET-OFF promoter, led to increased constitutive reporter expression following Dox repression. The authors reasoned that this behaviour could be explained by the reliance of both modules on the same finite pool of cellular resources. Hence, when one module is repressed by Dox induction, more resources become available for the other one to be expressed. In a paper by Jones et al.^8^ resources limitation in the context of transient transfections was probed using a genetic model system in which the GAL4 DNA binding domain (DBD) was fused to activation domains of different strength. By looking at the expression of a co-transfected constitutive reporter driven by different promoters, the authors proved that the stronger the activation by the synthetic transcription factor, the more resource demanding the synthetic device, and the sharper the decrease in the reporter expression. Importantly, these works show that, even if to a different extent, resource limitation is independent of the cell type and the promoter used, pointing to the need for a solution that can be generally applied to several cellular contexts.

If overlooked and not included in experimental analysis, the effects of resource competition, both at the level of the host cell and the construct, can represent a source of variability^9–12^.

In light of these observations, we are compelled to re-examine experimental co-transfection designs where resource competition may lead to data misinterpretation.

## Transient transfection in mammalian cell experiments

Transient transfection is used for the expression of recombinant proteins or prototyping synthetic gene circuits to avoid the lengthy procedures involved in stable cell line generation. On a higher level and beyond the synthetic biology field, transient transfections provide a general means to induce gene perturbation in fundamental biology experiments and to study the overall response of the observed systems. In contrast to stably integrated heterologous genes, transiently transfected genes are episomal and are therefore lost as cells proliferate. Further, transfection efficiency has a narrow optimal range, relying on DNA complex formation, solution pH, cell membrane condition and cell type^13^ and differences in transfection efficiency and DNA copy number may confound experimental results. Finally, individual cells from the same batch may exhibit different ability to produce recombinant protein^14^, adding additional variability. To avoid experimental biases originating from these sources of variability, researchers often use a transfection control for normalisation. This longstanding and widespread practice is similar to that described by Huliak et al., where a constitutively expressed reporter gene is co-transfected with the gene of interest either on a separate plasmid or as a second cassette within the same plasmid. The transfection control can serve as a marker for plasmid uptake within the population. It also provides confidence in data interpretation by facilitating normalisation of the expression of the gene of interest against a constitutively expressed reporter. By using the same transfection control with the test conditions, variability in transfection efficiency can be accounted for through the above normalisation procedure, allowing the user to capture the behaviour of interest.

## Drawbacks in the use of transfection controls

After transfection controls were adopted as a standard methodology, it became clear that there were issues with the practice. While some of the observed effects of co-transfection were linked to the specific constructs adopted in the experiments (e.g. interference between adopted promoter pairs, specific sequences that can respond to endogenous transcription factors, and presence of non-specific transcription events from the plasmid sequence imposing extra load and toxicity to the cells^15–17^), the fluctuation in the transfection control expression due to resource loading arose as a systemic effect to be taken into account^18–20^.

An example comes from the characterisation of promoter activity in mammalian cells. While determining the activity of putative DNA promoter sequences, Farr et al. noticed that the expression of the transfection control plasmid used to account for variability in transfection efficiency was heavily suppressed following co-transfection with the cognate positive control in HeLa cells^18^. The authors noted that this would result in overestimation of the promoter activity in the positive control compared to the test sequences. Similar problems can be envisioned when calculating the dose-response curve of inducible promoters, as displayed by Gyorgy et al. and Frei et al. Therefore, any normalisation against the transfection control will yield overestimated normalised expression values at high inducer concentrations.

The impact of misinterpreted data goes beyond the experiment it pertains to. Indeed, the current direction of synthetic biology and cell engineering toward standardisation^21^ and the ability to share genetic parts^22^ requires reliable characterisation of the behaviour of each component. Identifying methods for the in vivo characterisation of genetic parts which are not confounded by resource competition is necessary to enable the development of predictable designs for cellular engineering.

Considering the mounting evidence for resource competition highlighted above, we believe it is now time for a deeper debate focussing on the development of novel and improved approaches for the normalisation of biological data that are robust to resource competition.

## Routes toward more robust transfection controls

**Fig. 2:**
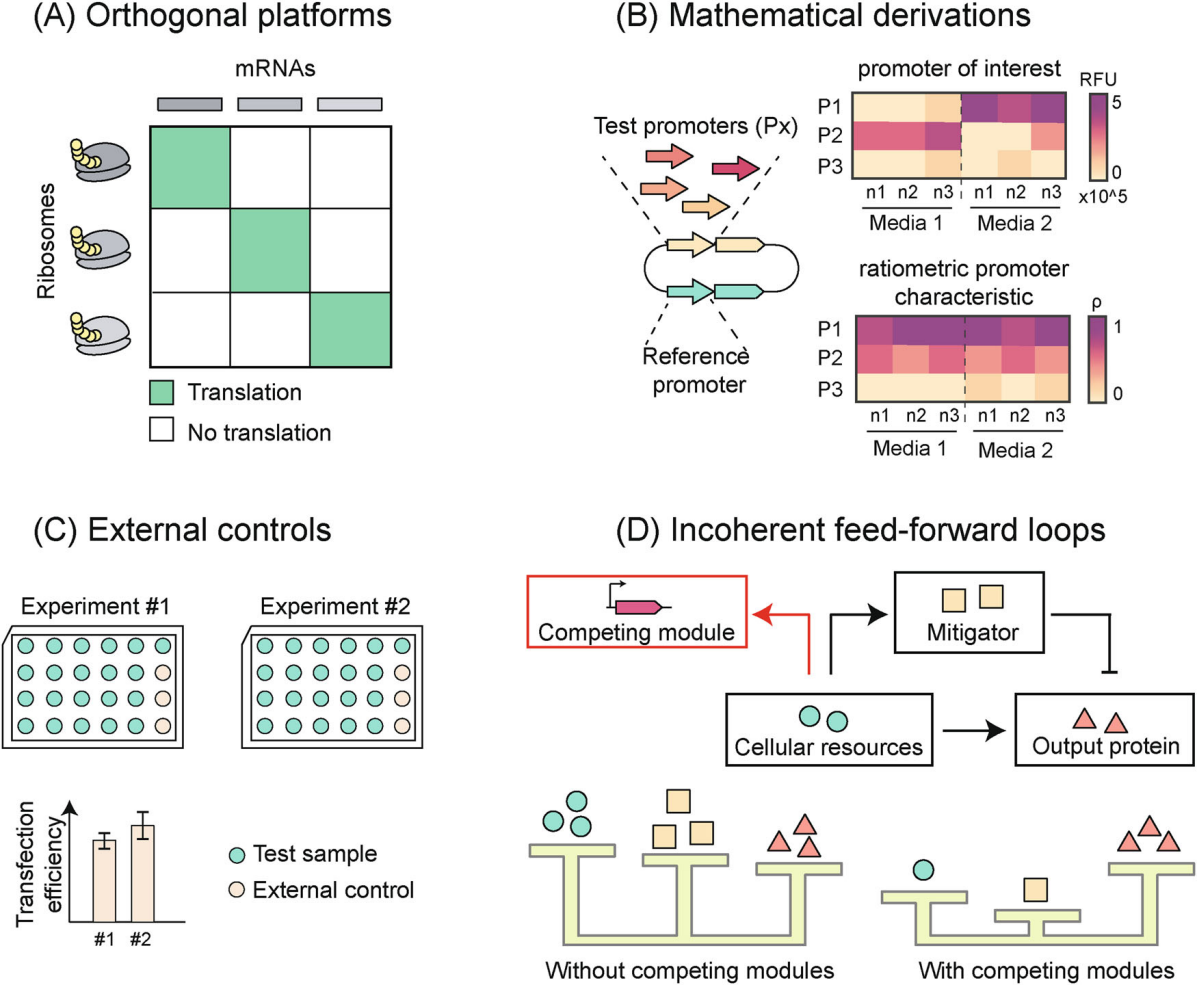
Alternatives to traditional transfection controls. **A** Platforms functioning orthogonally to the cell machinery have been developed. These designs are uncoupled to the cell machinery with the double advantage of both stable expression and minimal burden imposed on the engineered cells (adapted from Carlson et al.^25^). **B** Advanced mathematical derivations accounting for the global variation in part behaviour due to extrinsic factors allow precise definition of promoter characteristics (adapted from Rudge et al.^27^). **C** Resource loading following co-transfection can be avoided by using external reporters to control for inter-experimental variability in transfection efficiency (adapted from Brown et al.^31^). **D** Incoherent feed-forward loops (iFFLs), where cellular resources act as the input regulating the expression of both a mitigator and a mitigator-repressed output gene, are an effective way to buffer the output gene expression to fluctuations in the pool of cellular resources (adapted from Jones et al.^8^ and Lillaci et al.^6^).

We discuss below recent experimental and theoretical approaches that we believe can help address the problem (Fig. 2). We highlight their advantages and disadvantages and describe the scenarios in which they can be adopted. However, we do note that this is very much an open area of research in which potential novel solutions are yet to be discovered and existing alternatives are still limited.

## Orthogonal synthetic modules

One avenue to address this problem is the adoption of synthetic modules that are fully orthogonal to the cellular machinery, such as dedicated unnatural ribosomes that can only translate a specific set of mRNAs (Fig. 2A). This allows separation of resources for expression of orthogonal modules from expression of native genes and/or other co-transfected non-orthogonal constructs. Such designs have been demonstrated not only to provide stable and reliable expression, but also to reduce the burden imposed on the native machinery, thus representing a win-win approach that can prove useful to reduce experimental variability. Examples in bacteria from Cameron et al.^23^, Aleksashin et al.^24^ and Carlson et al.^25^ show how orthogonal protease-based or ribosome-based decoupling of circuit function can lead to controlled expression. Similarly, we speculate that other orthogonal components may prove beneficial, such as unnatural nucleotides that go on to generate polypeptides containing unnatural amino acids^26^, thus relieving the endogenous pool of natural precursors from feeding into the synthetic system. Although not fully implemented in mammalian cells yet, future developments in this direction may allow us to re-gain confidence in normalisation controls for transfection variability by providing modules that are more robust to resource loading than standard transfection controls. It must be noted, however, that using such orthogonal platforms reduces some, but not all, resource coupling, and further development in this direction is needed.

## Mathematical transformation of data

Mathematical transformation of the data can prove useful as well. For example, Rudge et al.^27^ introduced a ratiometric promoter characteristic (RPC) that is largely insensitive to extrinsic variability (i.e. expression variability due to differences in mRNA degradation, translation, fluorescent protein maturation and growth). They transformed *E. coli* with a plasmid bearing two constitutively expressed fluorescent reporters, one under the control of a test promoter (with a total of six promoters tested), the other under the control of a reference promoter. The authors observed a linear relationship between fluorescence intensity and biomass (absorbance) during the exponential phase for both promoters, showing how the slope of this relationship describes an intrinsic promoter characteristic (ap) that is largely insensitive to growth rate but still impacted by other factors (e.g. growth media) (Fig. 2B). Further, they demonstrated that normalising the ap of the test promoter to the ap of the reference promoter gave an RPC that was very robust to variations of extrinsic factors. This and similar mathematical approaches can be useful to reduce variance in measures of promoter activities due to extrinsic factors, thus representing a better alternative to the calculation of relative promoter units where promoter activity in different experimental conditions is normalised to a reference sample assessed in the same context^28,29^. This can lead to deeper precision when annotating part behaviour and is applicable to co-expressed systems, thus greatly benefitting the standardisation agenda. However, the major caveat of this method is that it relies on normalising the reporter expression by the growth rate in the exponential phase, which is feasible for bacterial systems, but difficult to apply to situations such as transient transfection where plasmids number is diluted with each cell division^30^. Advanced mathematical derivations better capturing variability in genetic part behaviour due to resource loading are awaited.

## External controls and resource-decoupled modules

Approaches that eliminate the use of controls entirely can also be envisioned. For instance, the adoption of transfection controls could be replaced by the use of external reporters to keep track of transfection efficiency variability and confirm construct behaviour with no need for normalisation (Fig. 2C). This is supported by Brown et al. who, in a recent study on the design of synthetic promoters in CHO cells, mention their choice to omit transfection controls to avoid promoter interference, opting instead for the use of separate samples transfected with constitutively expressed reporters with varying expression strength to assess the variability in transfection efficiency among experiments^31^. Although less elaborate, this approach is of immediate use, due to its simplicity. However, this approach relies on precise control over the transfection conditions to ensure that the experiments are not affected by variability in transfection efficiency as even small variations in cell state or plasmid DNA quality at the point of transfection can lead to large variations in the data.

Finally, the development of resource-decoupled modules to function as transfection controls can be envisioned. Previously, this strategy has been explored in microbial systems^32–35^. In mammalian cells, a method recently proposed is the incoherent feedforward loop (iFFL) circuit topology, which represents a core motif in biology and has been often used in synthetic circuits for its adaptation properties^6,8^. In iFFLs an input A regulates the expression of both an intermediate gene B (resource mitigator) and an output gene C, which is also negatively regulated by B (Fig. 2D). Jones et al. designed an endoribonuclease-based incoherent feed-forward loop (iFFL) where cellular resources act as the input A, regulating the Cas6 (B)-driven repression of the output gene C^8^. When cellular resource availability decreases (e.g., due the presence of competing modules), the expression of Cas6 diminishes, increasing the expression of the output gene. In this way, the decrease in the output expression due to resource limitations is buffered by the partial release of Cas6-driven repression, and the expression of the transfection control can be maintained as constant. An alternative to the protein-based iFFL described by Jones et al., is the use of microRNA-based iFFLs as shown in Frei et al. Here, the use of endogenous or synthetic microRNAs as intermediate gene B in the iFFL designs was demonstrated to stabilize the expression of the gene of interest while perturbing the expression of a second gene, in either a two or three reporter system. Both systems show better adaptation to genetic load and DNA copy number variation as compared to the open loop version (no IFFL) of the same devices, mitigating the drawbacks of resource competition. Although conceptually more complex than standard transfection controls, IFFLs where a reporter gene acts as output C can guarantee a more stable expression under resource loading and can return higher-quality data when performing normalisation analyses.

## Future directions

While the above-mentioned strategies are possible routes to undertake for mitigating resource competition, alternative and more optimal solutions to the resource-mediated coupling of expression systems in living cells need to be sought. To our knowledge, orthogonal modules have not been implemented in mammalian cells yet, and RPC mathematical derivations that are insensitive to resource loading are not available as of today. In contrast, iFFLs have been developed and characterised in mammalian cells and therefore may be the most immediate avenue to explore amongst those discussed above, even if they introduce complexity in the construct design. The exploitation of iFFLs also comes to a cost, namely the lower expression of the output gene C. While this should not be a problem in experiment where the downregulated gene is used as transfection control, this design may not be optimal in experimental settings seeking to maximize the iFFL-controlled output gene. Given that resource competition is just one of many mechanisms that cells use to couple and interlink their processes and pathways, a unique and easy solution to the problem may not be easily identified.

An overarching question is, how should the scientific community respond? We believe that a wider discussion within the molecular, cellular and synthetic biology communities is needed to evaluate the different approaches and propose possible alternatives. Further experimental and theoretical work aimed at identifying and quantifying the extent of interference between co-expressed systems and identifying novel strategies for resource-decoupled control of gene expression is also needed.

A possible direction for research might focus on the application of existing technologies to limit resource competition. For example, transcriptomic and proteomics approaches could be used to shed light on how resource allocation is regulated inside cells. This would not only provide a better understanding of the problem, but practically can also help us identify pathways that can be exploited for its mitigation. Similar approaches could be adopted for identification of more suitable transfection controls that overcome resource-coupled expression.

Another potential avenue may be to identify a set of genetic parts with lower footprint on the host resources. While *E. coli* construct design has benefitted from the development of automated tools taking into account the load imposed on the cellular machinery^36^, such tools are still lacking for mammalian cells, and would hugely benefit the design of synthetic constructs with lower resource demand to be used for mammalian cell engineering or foundational experiments. While such tools are being developed and knowledge generated for the more complex mammalian systems, some may also opt for less time-consuming strategies to mitigate resource competition. An example might be the use of fluorescent dyes to label intracellular plasmid DNA to characterise transfection efficiency and plasmid uptake and avoid the need for a reporter gene as the normalisation control^37,38^. We believe there is no unique solution to the problem and a common effort, based on novel experimental and theoretical strategies as well as transparent data sharing and discussion, can pave the way for identification of possible solutions and mitigation approaches.
